# Impacts of climate change on rice production in Africa and causes of simulated yield changes

**DOI:** 10.1111/gcb.13967

**Published:** 2017-12-12

**Authors:** Pepijn A. J. van Oort, Sander J. Zwart

**Affiliations:** ^1^ Africa Rice Center Bouaké Côte d'Ivoire; ^2^ Centre for Crop Systems Analysis Wageningen Universiteit Wageningen The Netherlands; ^3^ Faculteit Geo‐Informatie Wetenschappen en Aardobservatie Universiteit Twente Enschede The Netherlands

**Keywords:** Africa, climate change, cold induced sterility, heat induced sterility, irrigated, photosynthesis, rainfed, rice

## Abstract

This study is the first of its kind to quantify possible effects of climate change on rice production in Africa. We simulated impacts on rice in irrigated systems (dry season and wet season) and rainfed systems (upland and lowland). We simulated the use of rice varieties with a higher temperature sum as adaptation option. We simulated rice yields for 4 RCP climate change scenarios and identified causes of yield declines. Without adaptation, shortening of the growing period due to higher temperatures had a negative impact on yields (−24% in RCP 8.5 in 2070 compared with the baseline year 2000). With varieties that have a high temperature sum, the length of the growing period would remain the same as under the baseline conditions. With this adaptation option rainfed rice yields would increase slightly (+8%) but they remain subject to water availability constraints. Irrigated rice yields in East Africa would increase (+25%) due to more favourable temperatures and due to CO2 fertilization. Wet season irrigated rice yields in West Africa were projected to change by −21% or +7% (without/with adaptation). Without adaptation irrigated rice yields in West Africa in the dry season would decrease by −45% with adaptation they would decrease significantly less (−15%). The main cause of this decline was reduced photosynthesis at extremely high temperatures. Simulated heat sterility hardly increased and was not found a major cause for yield decline. The implications for these findings are as follows. For East Africa to benefit from climate change, improved water and nutrient management will be needed to benefit fully from the more favourable temperatures and increased CO2 concentrations. For West Africa, more research is needed on photosynthesis processes at extreme temperatures and on adaptation options such as shifting sowing dates.

## 1 INTRODUCTION

Africa is far from self‐sufficient in rice and this situation is projected to worsen in the future (Balasubramanian, Sie, Hijmans, & Otsuka, 2007; Van Ittersum et al., 2016; Van Oort, Saito et al., 2015). Keeping up with growing population and per‐capita rice consumption will require substantial yield gap closure and area expansion, or continued import dependency. Projections in these self‐sufficiency studies did not include climate change effects, which are still quite uncertain for rice in Africa. Possible climate change impacts on rice productivity have been simulated previously extensively for Asia (Aggarwal & Mall, 2002; Li et al., 2015; Matthews, Kropff, & Bachelet, 1995; Matthews, Kropff, Horie, & Bachelet, 1997; Tao, Hayashi, Zhang, Sakamoto, & Yokozawa, 2008). These studies suggested that future rice yield declines would be caused mainly by two factors: (i) increased heat induced sterility and (ii) shortening of the growing season. On both factors, significant advances have been made since the seminal works by Matthews in the 1990s. Earlier studies (Dingkuhn, Sow, Samb, Diack, & Asch, 1995) and later studies (Gaydon et al., 2017; Van Oort, De Vries, Yoshida, & Saito, 2015) cast doubt on the general validity of the heat sterility model in the ORYZA000 model version used by (Matthews et al., 1995, 1997), suggesting that the original heat sterility model overestimates heat sterility in hot (semi) arid climates. This led to the development of new canopy temperature models and heat sterility models for rice (Julia & Dingkuhn, 2013; Van Oort, Saito, Zwart, & Shrestha, 2014) and for other crops (Eyshi Rezaei, Webber, Gaiser, Naab, & Ewert, 2015; Webber et al., 2016). The Julia and Dingkuhn (2013) heat sterility model was incorporated in an improved version of ORYZA2000 (Van Oort, De Vries et al., 2015). This improved version predicted yields more accurately and with less bias in a hot (semi) arid region in Senegal (Van Oort, De Vries et al., 2015). This finding is highly relevant for Africa with a large area of irrigated rice cultivated along the Senegal river, the Niger river and the Benue river systems in the hot (semi) arid Sudanian and Sahel zone. This new heat sterility submodel generally predicts lower heat sterility and simulates two adaptive mechanisms of the rice plant to cope with heat: increased transpirational cooling at higher temperatures and earlier flowering (earlier in the morning, when it is cooler) to avoid heat. On the second major cause of future yield decline reported by Matthews et al., shorting of the growing season, significant and relevant advances have been made in rice phenology modelling. The ORYZA000 model version used by Matthews et al. (1995, 1997) assumed slower development above the optimum temperature of 30°C, while more recent experimental and modelling work suggests no delay in development rate above the optimum temperature (Van Oort, Zhang, De Vries, Heinemann, & Meinke, 2011; Zhang, Li, Yang, & Simelton, 2016; Zhang, Zhu, & Yang, 2008). Newer phenology models predict a stronger shortening of the growing season in the hotter climates and therefore more negative effects of climate change. These scientific developments on heat sterility modelling and phenology modelling call for new climate change studies with an improved crop growth model.

There have to date been no comprehensive climate change impact studies for rice in Africa as have been presented for Asia. (Sultan & Gaetani, 2016) reviewed climate change impact studies for West Africa. From this overview we can see that most climate change impact studies have been on other crops than rice. Another recent study on nine crops in Africa did not include rice because rice was considered a relatively small crop in Africa and because the model used could not simulate paddy rice (Rippke et al., 2016). We found only three recent published country specific studies on climate change on rice in Africa. Gerardeaux, Giner, Ramanantsoanirina, and Dusserre (2012) and Daccache, Sataya, and Knox (2015) both used the CERES‐Rice model and both found small positive effects of climate change on rice in Madagascar and Malawi respectively. Adejuwon (2006) predicted for Nigeria using the EPIC crop model that rice yields would increase with temperature changes up to +2/+3°C (*T*_max_/*T*_min_) and decrease with a +4/+5°C change. Two global studies, by Liu et al. (2008) and Lobell et al. (2008), estimated that rice yields in West and Central Africa would slightly decline and those in East and Southern Africa would slightly increase with climate change. The very limited number of studies, all with different models, does not yield a consistent or comprehensive estimate of climate change impact on rice in Africa. Here, we present a new study covering more countries and using one (improved) model throughout.

Crop growth models can be used as tools to quantify possible impacts of different climate scenarios. They integrate many effects and physiological interactions during the growing season. The manifold of processes included in these models sometimes makes it less clear why certain changes in yield emerge. Apart from using a crop growth model (ORYZA2000) to assess possible climate change impacts we also use the model in a diagnostic mode to explore the main causes of projected yield increases or declines.

The objectives of this study were to simulate climate change impacts on future rice production in Africa and explore causes of impacts. We used an adapted version of the ORYZA2000 model (Van Oort, De Vries et al., 2015) to simulate rice yields for irrigated systems in the wet and in the dry season and for rainfed systems for a typical lowland soil and a typical upland soil. We analysed yield changes for all four climate change scenarios comparing the 2000s with the 2070s. As an adaptation option for farmers we simulated the effects of farmers switching to varieties with a higher temperature sum. Temperature sum refers to the cumulative degree‐days which the crop requires to progress between phenological stages.

## 2 MATERIALS AND METHODS

### 2.1 Data

#### 2.1.1 Site selection

The ORYZA2000 crop growth model is a point‐based model, i.e. it is used to simulate rice yields for specific sites and does not model landscape hydrology. Input data can be weather station data or time series of weather data for specific pixels of gridded weather datasets. We first selected countries to cover the main rice growing areas and to assure that both irrigated and rainfed production areas were well‐represented. Selected sites within each country refer to individual pixels of 0.25^°^ spatial resolution, the spatial resolution of the weather dataset. Pixels were selected such that they were located centrally in major rice regions and climate zones of selected countries (Grassini et al., 2015; Van Bussel et al., 2015; Van Wart et al., 2013). Table 1 lists the countries for which simulations were conducted and the number of sites simulated per country for irrigated and rainfed rice. Together, rice harvested area (based on SPAM2005; You, Wood, Wood‐Sichra, & Wu, 2014) of these countries represent 74% of Africa's rice harvested area. According to the SPAM2005 data set, 26% of Africa's harvested rice area is irrigated, whereas in our dataset, the share of irrigated rice is slightly higher (32%).

**Table 1:** Rice harvested areas and number of simulation sites for countries in this study

Region^a^	Country	Irrigated area^b^ (1,000 ha)	Rainfed area^b^ (1,000 ha)	Irrigated sites^c^	Rainfed sites^c^
WEST	Benin	13	15	1	1
	Burkina Faso	21	28	3	4
	Ivory Coast	24	331	6	8
	Cameroon	26	22	1	0
	Ghana	13	108	2	3
	Gambia	2	14	1	1
	Mali	268	160	4	3
	Mauritania	17	0	1	0
	Niger	0	22	4	0
	Nigeria	25	2,493	6	9
	Senegal	41	48	2	0
NORTH	Egypt	643	0	1	0
EAST	Ethiopia	0	6	0	1
	Kenya	17	1	1	0
	Madagascar	910	348	15	13
	Rwanda	6	6	1	0
	Tanzania	0	649	4	3
	Uganda	10	97	0	5
	Zambia	3	9	0	1
Total	Countries simulated	2,040	4,356	53	52
	Africa total	2,280	6,401		

a The classification into West, North and East is ours, based on geographic location which roughly also corresponds with growing conditions (East is cooler than West; North is semiarid, warmer than East but cooler than West).

b Total harvested area based on SPAM2005 (You et al., 2014). Note if in a country two rice crops are harvested per year, then harvested area is 2× the physical area. Especially in irrigated systems double rice cropping is found.

c The last two columns show the total number of sites (point locations central in key rice producing areas) used in the simulations.

#### 2.1.2 Climate change data

We simulated four climate change scenarios (Representative Concentration Pathways RCP2.6, 4.5, 6.0 and 8.5; Van Vuuren et al., 2011) that differ in trend of CO_2_ and temperature. We used downscaled climate change scenario data (Ramirez & Jarvis, 2008) made available by the GCM Downscaled Data Portal (www.ccafs-climate.org). We downloaded maps of maximum and minimum temperature for time slices of the 2000s and 2070s, for the four RCPs and 34 general circulation models (GCM), at the highest spatial resolution available (2.5 arc min). A listing of the available data per GCM and scenario is provided in Table S1. In summary, we downloaded a total of 954 downscaled climate maps of *T*_max_ and *T*_min_. Temperature maps were overlaid with maps of rice cultivated areas as identified by the MIRCA dataset (Portmann, Siebert, & Doll, 2010). We then calculated seasonal average *T*_max_ and average *T*_min_ over the months included in the growing seasons and over the GCM's available. For this purpose we used the growing seasons defined in the RiceAtlas database (Laborte et al., 2017). Seasonal changes (2000s to 2070s) in *T*_min_ and *T*_max_ were then added to the daily weather data. The full procedure is outlined in detail in (Zwart, 2016) which also provides a link to the downloadable set of maps of changes per season and scenario for *T*_max_ and *T*_min_.

**Figure 1:**
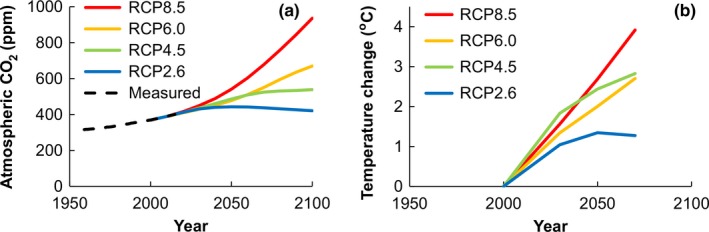
CO_2_ and temperature scenarios. (a) Projected changes in atmospheric CO2 concentrations in the 4 RCP scenarios and (b) projected temperature changes averaged over the study sites in the main growing season

**Figure 2:**
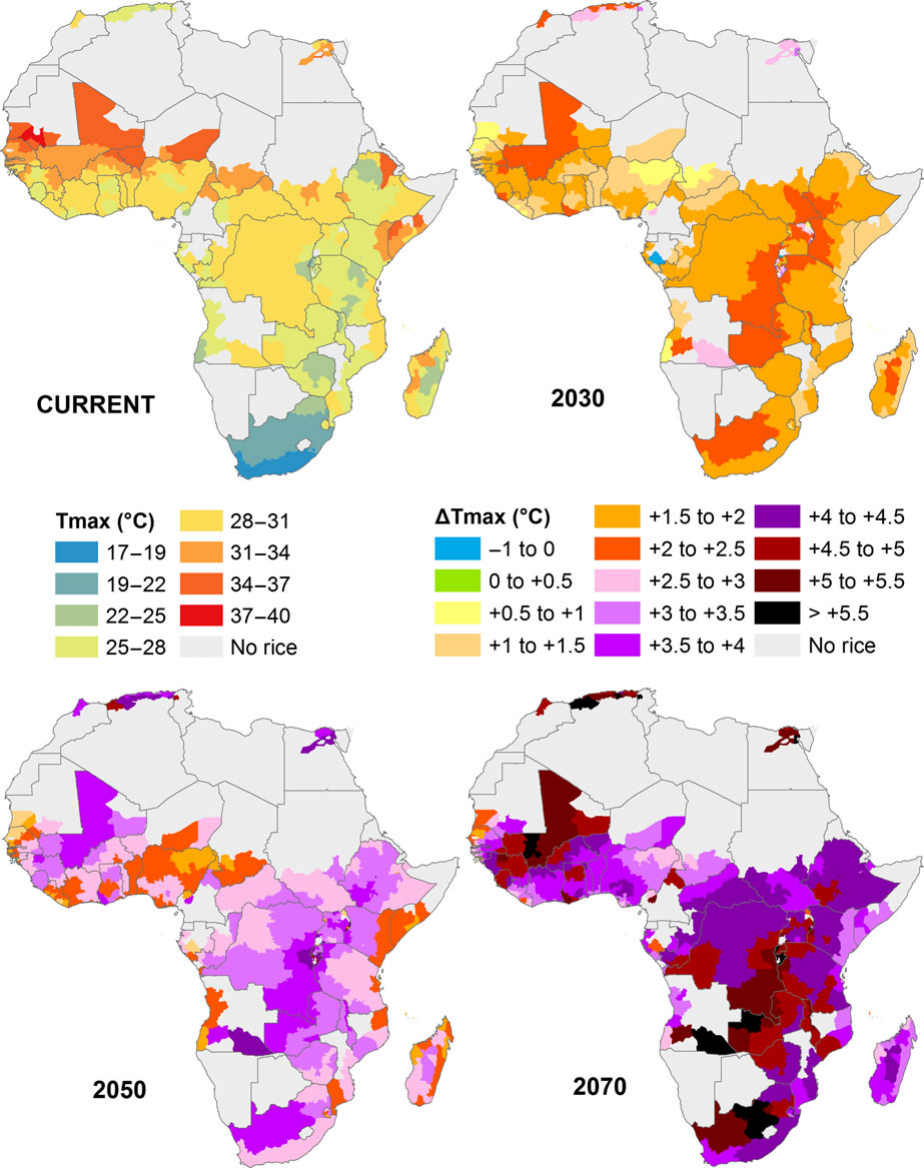
Projected changes in maximum temperatures from 2000 (current) to 2070 in RCP scenario 8.5 for rice growing areas in the main growing season

The CO_2_ trends were assumed to be the same across the continent (Figure 1a) and derived from (Prather et al., 2013). Figure 1b shows average temperature changes for rice in the main rice growing season, averaged over the growing seasons and sites used in this paper. Figure 2 shows spatial variability in projected temperature changes for *T*_max_ in the most extreme scenario. Consistent with most climate change scenarios future relative humidity was assumed to remain unchanged. Possible changes in rainfall patterns were not simulated because uncertainty in future rainfall projections is too large (Giannini, Biasutti, Held, & Sobel, 2008; Lobell & Burke, 2008; Lobell et al., 2008).

#### 2.1.3 Weather data

For selected sites we used daily weather data from the AgMERRA dataset, which contains daily weather data for crop modelling from 1980 to 2010 at 0.25 degrees spatial resolution (Ruane, Goldberg, & Chryssanthacopoulos, 2015). Many climate change impact studies have been using as a baseline a period in the past and not recent climate (White, Hoogenboom, Kimball, & Wall, 2011). Forced by circumstances we also did so here, because the AgMERRA dataset does not include data of the last 7 years and because the baseline year of the climate change scenario data was the year 2000. For irrigated sites we simulated for 5 years (1998–2002) centred around the year 2000, the baseline year of the RCP data. For rainfed sites we simulated for 9 years centred around the year 2000; for the rainfed rice we used more years to overcome chance selection of a few extremely wet or dry years. Seasonal temperature change data were added to these daily time series and specific CO_2_ values for each time slice and scenario (Figure 1) were used in the simulations.

#### 2.1.4 Cropping calendars

Cropping calendar data were obtained from the RiceAtlas (Laborte et al., 2017). They were cross‐checked and locally adjusted where necessary using data collected by AfricaRice and national partners. For irrigated systems we simulated with the same average sowing date for each year. For rainfed systems this is not appropriate considering large interannual variabilities that exist in the starting dates of the wet season (Sultan, Baron, Dingkuhn, Sarr, & Janicot, 2005). For rainfed systems, we searched for the first suitable sowing date within a ±30 days window around the average sowing date (from the RiceAtlas, possibly locally adjusted based on our data). A day was considered suitable if cumulative rainfall over the preceding 7 days was larger than 20 mm. This criterion was chosen based on a previous study by (Wolf, Ouattara, & Supit, 2015). This sowing rule has not been calibrated with farmers’ data and one may expect that it differs locally depending on local hydrological and climatic conditions but as a general rule of thumb we expect it to be more accurate than simply assuming a fixed sowing date.

Crop development rates were calibrated such that, averaged over the simulated years, simulated duration from sowing to maturity equalled actual duration from sowing to maturity according to the RiceAtlas. More details about the phenology model and its calibration are found in section 2.2.3 of this paper.

#### 2.1.5 Simulations for irrigated and rainfed sites

For Asia (Matthews et al., 1995, 1997) simulated the possible changes in potential production, which is logical in Asia with its’ predominance of irrigated systems. According to (Balasubramanian et al., 2007) rice in Sub‐Sahara Africa is cultivated in four ecosystems: dryland (38% of the cultivated rice area), rainfed wetland (33%), deepwater and mangrove swamps (9%), and irrigated wetland (20%). Similar numbers can be obtained from Table 1. For irrigated sites we simulated potential production, for rainfed rice we simulated water limited production. Both presume ample nutrient supply and no major problems of weeds, pests, diseases and soil toxicities. The only difference is that in water limited production, effects of drought are also simulated while under potential production the assumption is that no drought stress occurs (Bouman et al., 2001).

Rainfed rice yields depend strongly on groundwater depths for which there exist no reliable data at a continental scale and high temporal resolution. We therefore made two assumptions, using the following two soil types:

- Rainfed lowland rice on a clayey soil, not puddled, no plowsole, 25 cm high bunds, low percolation rate, groundwater constant at 40 cm depth;
- Rainfed upland rice on a sandy soil, not puddled, no plowsole, no bunds, high percolation rate, freely draining.

Associated soil parameters are listed in the Supporting Information. The absence of a plowsole is common in African rice systems. Puddling is rare. Bunding is frequently applied in lowland soils and rarely in upland soils. Percolation rates in the upland soils are often so high that any standing water would be lost anyway, whether through run‐off (in case of no bunding) or percolation (with bunding). Lowland soils typically have more clay and lower percolation rates, caused by sediment transport over the millennia and low hydraulic head in inland valley bottoms and flood plains. There is a continuum in the landscape position from the completely freely draining soils higher uphill towards the valley bottoms (Danvi, Jütten, Giertz, Zwart, & Diekkrüger, 2016; Schmitter, Zwart, Danvi, & Gbaguidi, 2015; Windmeijer & Andriesse, 1993). With the two soil types here we simulated two contrasting positions in this continuum.

### 2.2 ORYZA2000: recent improvements

The ORYZA2000 model (Bouman et al., 2001) was developed in the 1990s. Since around 2010, improvements have been made in three different versions of this original model. At the international rice research institute (IRRI) ORYZAv3 was developed as a successor of ORYZA2000 (Li et al., 2017). The physiological part of ORYZA2000 (Bouman et al., 2001) has been integrated into APSIM, i.e. the “IRRI” rice plant is grown on top of an “APSIM” soil (Gaydon et al., 2012). Uncertainties and recent improvements are discussed in (Li et al., 2017) for ORYZAv3 and in (Gaydon et al., 2017) for APSIM‐ORYZA. Improvements by these two groups involved more accurately modelling soil processes (water × N interactions, soil carbon, root growth), multicrop sequences, effects of salinity on crop physiology (Radanielson, Angeles, Li, Ismail, & Gaydon, 2017) and interaction effects of water and N limitation on crop physiology. At the Africa Rice Centre, where the authors of this paper are employed, improvements have been made on heat sterility, cold sterility and phenology (Van Oort, De Vries et al., 2015). The latter are of particular relevance in the context of climate change studies. Some new improvements are presented below. The new version used here is called ORYZA2000v2n14s1, which builds on the ORYZA2000v2n13s14 version (Van Oort, De Vries et al., 2015) which in turn is based on the original ORYZA2000 model (Bouman et al., 2001).

#### 2.2.1 Heat sterility

Research on heat induced sterility in the last decades has shown that rice has two natural mechanisms to avoid heat sterility (Julia & Dingkuhn, 2012, 2013; Matsui, Kobayasi, Yoshimoto, & Hasegawa, 2007; Van Oort et al., 2014; Van Oort, De Vries et al., 2015). Firstly, transpirational cooling of the spikelets increases at higher temperatures and higher vapour pressure deficit and is therefore especially large in hot semiarid environments, where spikelet – air temperature differences as high as 12°C have been reported (Julia & Dingkuhn, 2013). Secondly, in hotter climates rice flowers open earlier in the morning, when temperatures are still lower, thus avoiding the hottest part of the day (Julia & Dingkuhn, 2012). (Van Oort, De Vries et al., 2015) showed that the heat sterility submodel in ORYZA2000 (Bouman et al., 2001) which does not account for these two mechanisms, grossly overestimates heat sterility in hot semiarid irrigated systems in Senegal. A recent study suggests that ORYZA2000 is also overestimating heat sterility in hot semiarid parts of Asia (Gaydon et al., 2017). The ORYZA2000v2n14s1 used in the current paper explicitly simulates transpirational cooling and earlier flowering in hotter climates (Van Oort, De Vries et al., 2015).

#### 2.2.2 Cold sterility

Cold is relevant because in parts of Africa rice suffers from cold induced sterility and this may decrease in the future with temperature rise. In Asia, Balwinder‐Singh, Yadav, and Gaydon (2015) kept the original ORYZA2000 cold sterility equation but changed the threshold temperature for cold induced sterility from 22 to 28°C, which suggests that the default equation underestimated cold stress. For Africa in Senegal (Dingkuhn & Miezan, 1995; Dingkuhn et al., 1995) and later (Van Oort, De Vries et al., 2015) changed the original cold sterility equations of ORYZA2000, because ORYZA2000 underestimated cold sterility. A recent study showed that this new cold sterility model is still unable to adequately simulate across different environments. Dingkuhn et al. (2015) showed that for the same variety at the same estimated minimum water temperature at booting to heading stage, measured cold sterility was much larger in Senegal than in Madagascar. The nature of cold is different in East and West Africa. In West Africa cold occurs mainly in irrigated rice systems in the Sahel zone just south of the Sahara desert with large diurnal temperature amplitudes, requiring tropical varieties adapted to high daytime temperatures, which may be less adapted to night time cold. In East Africa cold occurs in the highlands with prolonged cold and smaller diurnal amplitudes, and varieties growing in this cooler climate may be better adapted to cold, perhaps through some yet poorly understood process of acclimation. This roughly explains the greater cold sensitivity in West Africa. We used for West and East Africa the following equations derived from data reported in (Dingkuhn et al., 2015) for variety IR64:

(1a) $$SFCOLD1=\left\{\begin{array}{cc} \max(0,\min\left(1,1-\left(2.32-0.104\times T_{\mathrm{wmin}}\right)\right)) & \mathrm{WEST}\\ \max(0,\min\left(1,1-\left(1.04-0.046\times T_{\mathrm{wmin}}\right)\right)) & \mathrm{EAST} \end{array}\right.0.65\leq DVS<0.825$$

(1b) $$SFCOLD2=\left\{\begin{array}{cc} \max(0,\min\left(1,1-\left(2.32-0.104\times T_{\min}\right)\right)) & \mathrm{WEST}\\ \max(0,\min\left(1,1-\left(1.04-0.046\times T_{\min}\right)\right)) & \mathrm{EAST} \end{array}\right.0.65\leq DVS<0.825$$

(1c) $$\mathrm{SFCOLD}=\min\left(\mathrm{SFCOLD}1\right)\times \bar{SFCOLD2}$$

Following (Dingkuhn et al., 2015; Julia & Dingkuhn, 2013) and (Van Oort, De Vries et al., 2015) we simulated from development stage (DVS) 0.65 to 0.825 (microspore phase) with minimum water temperature *T*_wmin_ (Equation 1a). From DVS 0.825 to 1.0 (panicle exertion phase) we used daily minimum air temperature *T*_min_ (Equation 1b), because during this phase the cold sensitive meristem is above the water level. Final cold fertility SFCOLD (Equation 1c) was calculated as the minimum value of SFCOLD1 from DVS 0.65 to 0.825, multiplied with the average value of SFCOLD1 from DVS 0.825 to 1. The rationale for taking the minimum and average is explained and tested in Van Oort, De Vries et al. (2015). The empirical equation for estimating minimum water temperature is found in Van Oort, De Vries et al. (2015) and is based on original work reported in (Julia, 2012).

#### 2.2.3 Phenology

ORYZA2000 simulates phenology at an hourly timestep and integrates over the day. The ORYZA2000 version used by (Matthews et al., 1995, 1997) contained a phenology model in which development rate increases above the base temperature of 8°C and then decreases again above the optimum temperature of 30°C, with no development above the maximum temperature of 42°C. Later studies showed that models with no delay in development above the optimum temperature generally simulate the length growing period more accurately (Van Oort et al., 2011; Zhang et al., 2016). These studies showed that in tropical environments the base temperature is often higher than 8°C. Use of a wrong phenology model can cause significant bias in yield simulations (Van Oort, De Vries et al., 2015; Zhang et al., 2008). After fixing the cardinal temperatures (see below) and assuming no photoperiod sensitivity we calibrated development rates for each site and season such that simulated duration from sowing to maturity would match values reported in the RiceAtlas. The 50% flowering date was fixed to occur 30 days before the maturity date (Vergara & Chang, 1985) in the current climate. In this paper we simulated with a bilinear temperature response phenology model with a base temperature of 14°C, an optimum temperature of 31°C and no delay in development at temperatures above 31°C, with values of 14°C and 31°C based on (Van Oort et al., 2011) and (Sanchez, Rasmussen, & Porter, 2014).

#### 2.2.4 Other modifications

Two other modifications were made relative to the ORYZA2000v2n13s14 version (Van Oort, De Vries et al., 2015) and relevant in the context of climate change. Firstly, the original ORYZA2000 model did not allow for simulating separately the effects of increasing daily minimum and maximum temperatures. Climate change studies consistently show that minimum temperatures increase more than maximum temperatures. We built into the model the option to separately increase *T*_min_ and *T*_max_. Secondly, we corrected the method of simulating future change in humidity. The ORYZA2000 model reads daily actual vapour pressure from a weather file. If temperatures are increased, saturated vapour pressure increases. Without simultaneously increasing actual vapour pressure, the original ORYZA2000 predicts a decreasing relative humidity (RH). According to most climate change scenarios this will not happen, they predict RH will remain the same as in the current climate (Allen & Ingram, 2002; Held & Soden, 2000; Willett, Jones, Gillett, & Thorne, 2008). We found that simulations with decreasing relative humidity (as in the original model) led to large overestimation of future drought. To correct for this effect we incorporated a function that maintains the same level of RH between current climate conditions and climate change scenarios by adjusting the actual vapour pressure as needed.

#### 2.2.5 Model credibility

A number of steps were taken to establish model credibility. In the discussion we reflect on credibility of simulated yields under climate change. In this section we reflect on credibility of simulated yields for the current situation. A recent study has shown that a very similar model as used in the current study (APSIM soil, ORYZA2000 crop) performed well in a wide range of contrasting environments in Asia (Gaydon et al., 2017). This provides some confidence that the same model, with the improvements presented here, will also predict accurately in a wide range of environments in Africa. A validation of the ORYZA2000v2n14s1model for Senegal, with the new heat, cold and phenology model was presented in (Van Oort, De Vries et al., 2015) and showed a high model efficiency for this particular environment. We simulated potential production (irrigated environments) and water‐limited production (rainfed environments) which assumes no soil fertility limitations and no biotic and abiotic production constraints such as weeds, pests and diseases, salinity and iron toxicity. In reality, many of these conditions are present virtually everywhere in rice in Africa (e.g. Niang et al., 2017; Tanaka et al., 2017). Even the farmers with 10% highest yields (Tanaka et al., 2017) have yield gaps. Consistent with this, simulated potential yields were equal to or higher than these top10% yields (result not shown). This is logical but unfortunately still tells us little about whether these potential yield estimates are accurate. There are few quantified data available that can be used to validate potential yields. Therefore, two qualitative methods were applied to assess model credibility. Firstly, we checked if the model correctly simulated spatial patterns across climatic zones and production systems. This was indeed the case. Both model and data reported in Niang et al. (2017) and Tanaka et al. (2017) show highest yields in irrigated systems in the semiarid zone, where radiation levels are high and where, if irrigation and fertilizer are available, high yields can be obtained. Simulated and actual yields were also high in the main season in the highlands of East Africa (e.g. central Madagascar), because low temperatures lead to a long growing season, allowing for high biomass accumulation. Simulated and actual yields are lower in the rainfed systems, due to drought and interannual variation in simulated rainfed rice yields was larger than that of irrigated rice yields. The second qualitative model credibility check was expert consultation. In the global yield gap atlas (GYGA) project, credibility of 81 simulated yields for was assessed for eight African countries also included in the current study. Model credibility was assessed by agronomists of each country involved, based on their “expert knowledge” and in collaboration with a senior rice agronomist at the Africa Rice Center (K. Saito; see Van Oort, Saito et al., 2015). Country agronomists checked sowing dates, crop duration, yield levels and spatial patterns within their countries. This lead to various model improvements and this iterative process was continued until credible results were obtained for all sites, but unfortunately it did not result in reliable data for quantitative validation. Model accuracy also depends on quality of the input data. Quality controls as defined in (Grassini et al., 2015) were applied.

### 2.3 Causes of yield decline

We used the ORYZA2000v2n14s1 model to identify the main causes of future yield changes under climate change. In the model an elevated CO_2_ concentration has an exclusively positive effect on rice productivity. In the model air temperature affects rice growth and productivity in various ways (see Table 2 for an overview). From our simulation results two hypotheses emerged on which of the processes listed in Table 2 were the main causes of simulated yield decline. The following two sections describe how we tested these hypotheses.

**Table 2:** Effects of temperature rise on processes simulated in ORYZA2000

Process	Environment^a^	
	Cool	Hot
Early leaf growth	+	+
Respiration	+	+
Assimilation	+	−
Cold sterility	−	0
Heat induced sterility	0	+
Length growing period	−	−
Evapotranspiration	+	+

a a + means the process is increased, a − means it is decreased and a 0 means no change. *A* + does not per definition mean a positive effect on yield. If heat induced sterility increases or if respiration increases, then yield can decrease.

#### 2.3.1 Phenology

Development is faster at higher temperatures, leading to a shorter growing period and accordingly less time for accumulating biomass, hence lower yields. To investigate how much of the simulated yield loss was caused by shortening of the growing season we simulated scenarios in which growth duration would remain the same as in the year 2000. This analysis serves, firstly, to identify shortening growth duration as a cause of yield decline. Secondly, it can be considered an adaptation option for African rice farmers, showing what would happen if they would gradually replace their current varieties with new varieties that have a higher temperature sum, keeping pace with shortening of the season due to temperature rise. In the context of climate change, current varieties will have a shorter duration in the future and adapted varieties will have an unchanged duration (relative to the baseline year 2000) in the future.

#### 2.3.2 Effect of temperature on photosynthesis

**Figure 3:**
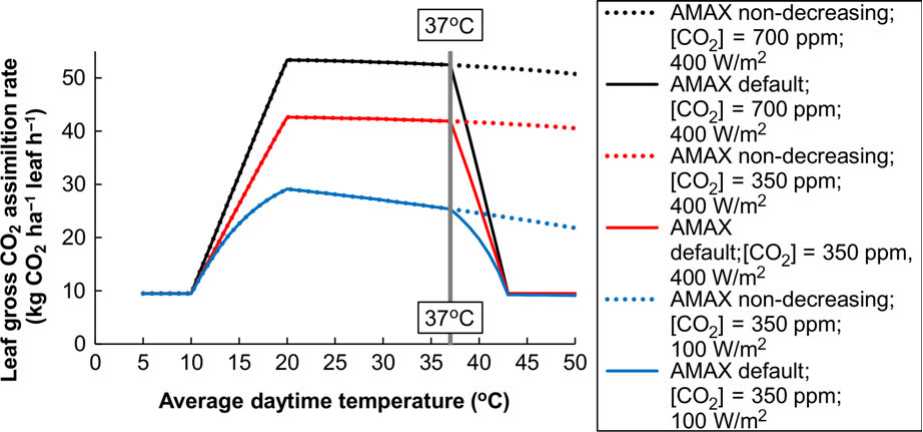
Leaf gross assimilation as affected by various factors in the ORYZA2000 model. The solid lines show the default response curves in ORYZA2000 at 1.5 g N/m^2^ leaf area at two atmospheric CO_2_ contents and two intercepted radiation levels. Average daytime temperature on the *x*‐axis is calculated as 0.75 × *T*_max_ + 0.25 × *T*_min_. The default shows a sharp decline in assimilation above 37°C. The dashed lines (AMAX nondecreasing) show a scenario explored in the current paper to investigate if simulated yield declines were caused by decreasing AMAX above 37°C daytime temperature. The small minimum assimilation rate of 10 kg CO_2_/ha leaf per hour below 10°C and above 43°C, is practically inconsequential

ORYZA2000 simulates leaf photosynthesis at three times of the day (at different temperatures and radiation levels early morning, midday and late afternoon) and at three canopy depths and integrates over the canopy and the day to obtain the daily gross assimilation rate (Bouman et al., 2001). Leaf photosynthesis in the model depends on leaf Nitrogen (N) content, intercepted radiation, atmospheric CO_2_ concentration and air temperature (Figure 3 and Supporting Information). The daily maximum assimilation rate AMAX (kg CO_2_/ha leaf per hour) is multiplied with a trapezoid shaped temperature response function with a base at 10°C, optimum from 20 to 37°C and declining sharply as daytime average temperatures increase from 37 to 43°C (solid lines in Figure 3). We tested if projected yield declines were caused by the sharp decline in AMAX from 37 to 43°C, by including a scenario in which AMAX was nondecreasing above 37°C (dashed lines in Figure 3). It should be noted that virtually no experimental data have reported from this temperature range. If model outcomes show great sensitivity of simulated yields to this temperature response, in specific areas, then those areas can be targeted for further experimentation.

## 3 RESULTS

### 3.1 Baseline yields

Table 3 shows average baseline simulated yields around the year 2000, with in brackets the standard deviation, showing spatial and temporal variation. Potential yields are higher in irrigated systems than in rainfed systems and interannual variability is less in irrigated systems. Simulated rainfed yields are substantially lower in rainfed upland than in rainfed lowland, which is consistent with lower water availability in the upland soils. Variation in yields is higher for rainfed systems than for irrigated systems, which is consistent with greater risk caused by drought.

**Table 3:** Baseline crop potential yields and variability

	East Africa	West Africa
Irrigated ‐ main season	7.8 (1.3)	8.1 (1.0)
Irrigated ‐ “off” season	6.7 (2.2)	7.2 (1.3)
Rainfed lowland (all seasons)	7.4 (2.8)	7.5 (2.0)
Rainfed upland (all seasons)	4.7 (2.7)	4.4 (2.6)

Yields shown in this table are averaged over 22 irrigated sites in East Africa (1998–2002), 31 irrigated in West Africa (1998–2002), 27 rainfed sites in East Africa (1996–2004) and 29 rainfed in West Africa (1996–2004). Yields are shown as potential yield *Y*_p_ (irrigated) and water limited yield *Y*_w_ (rainfed), in tonnes dry matter per hectare unmilled rice. In brackets is the standard deviation showing the interannual and site variability in tonnes dry matter per hectare. Rainfed rice is mostly grown in the wet season; in the central highlands of Uganda and Rwanda with bimodal rainfall patterns two seasons are found.

In many parts of the world dry (off) season irrigated crops yield more than wet (main) season irrigated crops, due to higher radiation levels in the dry season. Here the opposite was found, with on average lower potential yields in the dry (off) season. In West Africa this was caused by reduced assimilation at high temperatures in 16 hot inland irrigation systems along the Niger river (Mali, Niger, northern Nigeria, northern Benin) and the Benue in North East Nigeria. For the seven cooler irrigation systems in the coastal regions we did find higher simulated yields in the dry season (Mauritania: Rosso, Senegal: Ndiaye and Fanaye, Gambia: Sapu, Ghana: Yendi, Nigeria: Bida and Lokoja). This finding suggests that much of the dry season irrigated rice in the Sudano Sahel zone is around the 37°C tipping point (Figure 3) for which with further temperature rise dry season rice yields will decrease. Shifting rice sowing dates more into the cold dry season could be an adaptation option but might create logistic problems and human nutritional problems, because farmers now often use the cold dry season for growing vegetables (Van Oort et al., 2016). In East Africa irrigated systems simulated dry season yields were lower than wet season yields because most of the simulations for dry season irrigation systems were for sites in the cold dry winter season in Madagascar, where cold is negatively affecting potential rice yields. This finding suggests that in the future with temperature rise, dry season irrigated rice in Madagascar will become an increasingly attractive option.

### 3.2 Regional climate change impacts and causes

Yields from 2000 to 2070 are projected to decrease in all scenarios and environments (Table 4). Average yield decline is lowest in RCP scenario 2.6 (−9%) and highest in RCP scenario 8.5 (−24%). In the scenario with adaptation (varieties with higher temperature sum, “unchanged duration”) yields slightly increased (+5% in RCP scenario 2.6, +8% in RCP scenario 8.5). Therefore, it can be concluded that shortening of the growing period is the main cause of projected yield decline and this effect is present in all sites and all growing environments. Once we account for the adaptation option referred to as “unchanged duration” we find systematically different responses to climate change depending on rice growing environment and region:

**Table 4:** Average relative rice yield changes from 2000 to 2070

Water supply	Environment	Africa	RCP2.6		RCP4.5		RCP6.0		RCP8.5		
			Shorter duration (%)	Unchanged duration (%)	Shorter duration (%)	Unchanged duration (%)	Shorter duration (%)	Unchanged duration (%)	Shorter duration (%)	Unchanged duration (%)	Unchanged duration + AMAX nondecreasing (%)
Irrigated	Main	East	−10	+10	−18	+18	−14	+21	−22	+26	+26
	Season	West	−4	+5	−16	+5	−14	+7	−21	+7	+11
	“Off”	East	−9	+11	−17	+22	−13	+25	−20	+24	+24
	Season	West	−11	−4	−33	−19	−27	−13	−45	−15	+11
Rainfed	Lowland	East	−10	+6	−19	+4	−14	+7	−19	+7	
		West	−7	+4	−19	+3	−14	+4	−18	+7	
	Upland	East	−13	+2	−23	+1	−18	+2	−24	+5	
		West	−6	+5	−20	0	−15	+2	−22	+4	
Overall Average			−9	+5	−21	+4	−16	+7	−24	+8	+18

In the baseline simulations the duration (growing period) becomes shorter due to temperature rise. “Unchanged duration” is an adaptation option where the length of the growing period remains the same as in 2000, which would happen if farmers adapt to climate change by adopting varieties with a higher temperature sum, thus offsetting the shortening of the growing period due to temperature rise. For RCP8.5, we additionally simulated what would happen if maximum assimilation rate AMAX would not decrease at higher temperatures. For brevity this effect is only shown for RCP8.5. The overall average shown here is the average of the 8 rows above, not weighted by number of sites and therefore may differ slightly from the averages reported in Table 5. Rainfed rice is mostly grown in the wet season; in a few sites in the central highlands of Uganda and Rwanda with bimodal rainfall patterns two seasons are found.

- Rainfed rice yields increase less than irrigated rice (RCP8.5: upland: +4% to +5%, rainfed lowland +7%, irrigated +7 to +26%, exception is West Africa dry season, −15%)
- In three of four irrigated systems yields increase (RCP8.5: East Africa main season: +26%, East Africa off season +24%, West Africa main season: +7%)
- In West Africa irrigated rice in the dry season yields are predicted to decrease (RCP8.5: −15%)

**Figure 4:**
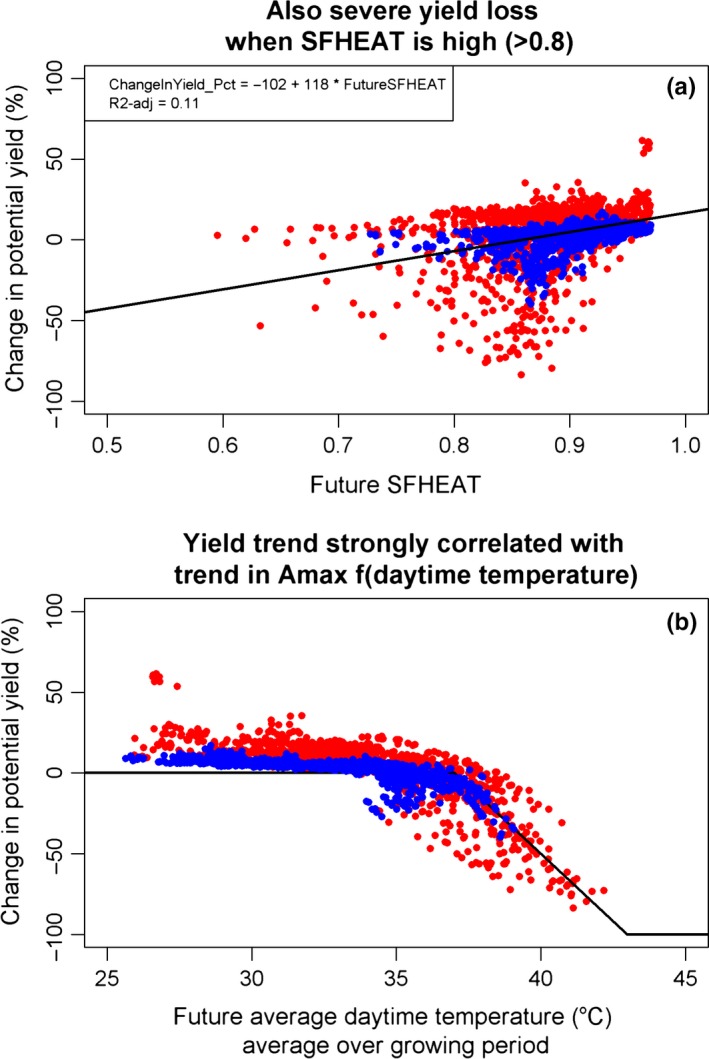
Simulated change in yields in irrigated sites in the “unchanged duration” scenario for RCP scenarios 2.6 (blue) and 8.5 (red). The top pane (a) shows on the *x*‐axis future spikelet fertility as affected by heat (SFHEAT, 0–1), which is 1 minus the spikelet sterility. In the bottom pane (b) the black line shows part of the trapezoid function of the temperature function with which the maximum assimilation rate AMAX is multiplied (Figure 3). AMAX is optimal (1, here scaled to 0%) from 20 to 37°C. From 37°C to 43°C, the temperature multiplier for AMAX drops to 0 (here −100%). Each dot represents a simulation for a site (53 irrigated sites Africa) in a specific season (main season or off season) and year (1998–2002)

The difference between rainfed and irrigated rice indicates that frequently during the growing season the main production limiting factor is water and not CO_2_ and therefore rainfed rice benefits less from CO_2_ fertilization. East Africa irrigated rice yields increase as a result of CO_2_ fertilization and because temperatures are often below optimum. In West Africa irrigated rice in the hot dry season yields decrease because of reduced assimilation. In the scenario with nondecreasing AMAX, the negative effect on West Africa dry season irrigated rice change (−15%) changed into a positive effect (+11%). Simulations suggested that average heat induced sterility would hardly increase. Apparently stronger transpirational cooling and earlier flowering opening times at elevated temperatures are enough to offset temperature rise, leading to almost unchanged spikelet temperatures at flowering opening time. Figure 4a shows that projected yield declines were not correlated with future spikelet fertility. Simulated yield declines were strongly correlated with the decline in maximum daily assimilation rate at daytime average temperatures above 37°C (Figure 4b).

### 3.3 Maps and stats by country

**Figure 5:**
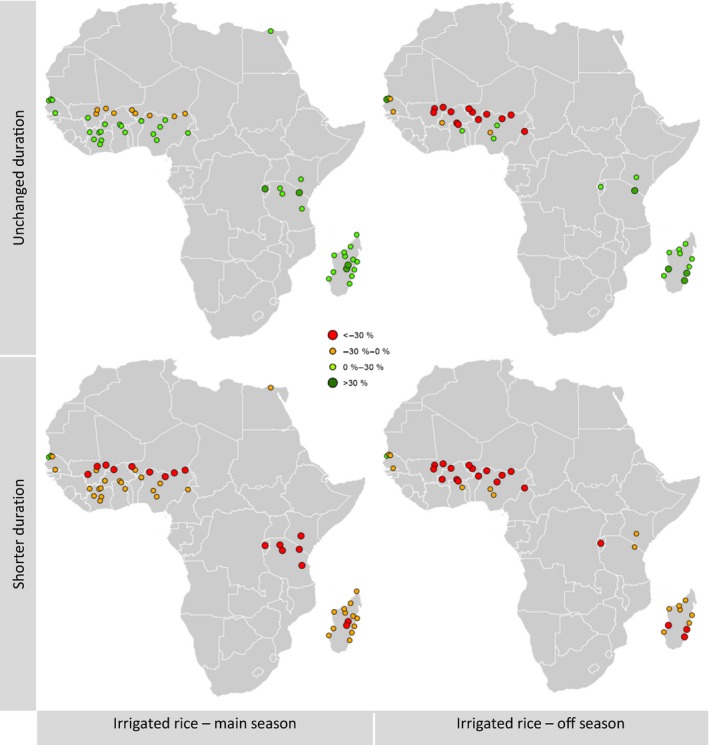
Irrigated rice climate change impact. Simulated changes in two seasons, with adaptation (“unchanged duration”) and without adaptation (“shorter duration”). For the main season and the off season. RCP scenario 8.5, changes 2000 to 2070

**Figure 6:**
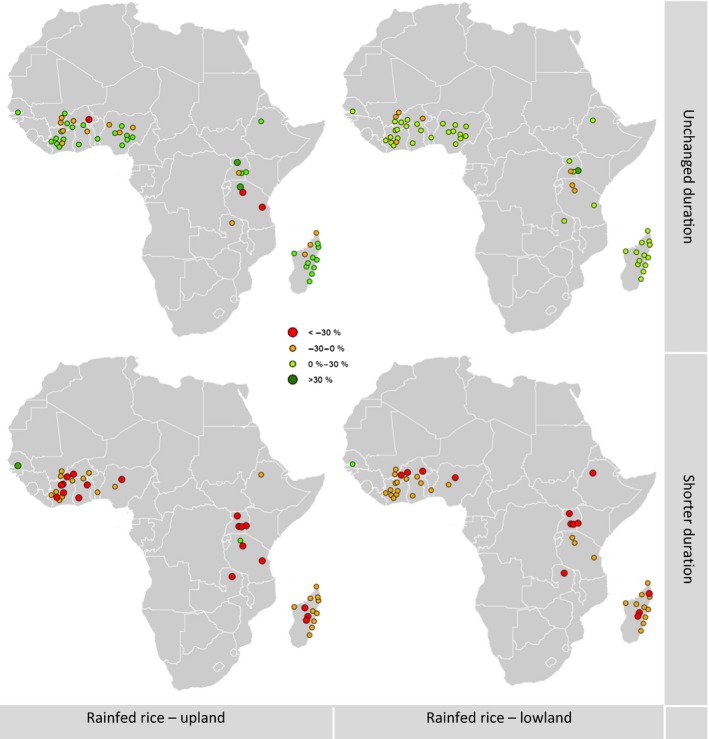
Rainfed rice climate change impact. Simulated changes in two seasons, with adaptation (“unchanged duration”) and without adaptation (“shorter duration”). For the main season, for two soil types/landscape positions. RCP scenario 8.5, changes 2000 to 2070

Table 5 shows the relative changes by country and environment for the most extreme scenario, RCP 8.5 in the year 2070. Figures 5 and 6 show the changes per site. The table as well as the maps show a gradient of yield decline in West Africa, with most severe yield decline in the hotter northern landlocked countries (Mali, Niger) and less in the cooler coastal countries of West Africa. In East Africa, all the cooler irrigated systems in the highlands (Kenya, Madagascar, Rwanda, Tanzania) benefit from climate change. For Egypt, for which we simulated only for the Nile delta, if varieties with unchanged duration are adopted, a small increase in yields (+6%) is simulated. Thus in Egypt yields do not increase as strongly as in the rest of East Africa (which is cooler) and do not decrease as strongly as in West Africa (which is hotter).

**Table 5:** Simulated changes in rice yield averaged by country and by environment

Africa	Country	RCP8.5, changes 2000 to 2070							
		Shorter duration				Unchanged duration			
		Irrigated		Rainfed		Irrigated		Rainfed	
		Main season (%)	Off season (%)	Low‐land (%)	Up‐land (%)	Main season (%)	Off season (%)	Low‐land (%)	Up‐land (%)
WEST	Benin	−13	−59	−22	−25	+13	−41	+13	+8
	Burkina Faso	−23	−48	−34	−32	+7	−32	+3	+10
	Cote D'Ivoire	−13		−13	−29	+17		+11	+7
	Cameroon	−4	−52			+14	−31		
	Ghana	−20	−36	−18	−35	+13	−16	+11	+2
	Gambia	−25	−30	+28	+85	+6	−5	+17	+25
	Mali	−33	−80	−10	−17	−7	−70	+2	+1
	Mauritania	+7	−14			+21	+2		
	Niger	−29	−45			−10	−47		
	Nigeria	−30	−42	−26	−28	+6	−18	+10	+2
	Senegal	4	−4			+18	+17		
WEST	Total	−20	−42	−19	−25	+7	−26	+9	+5
NORTH	Egypt	−19				+6			
EAST	Ethiopia			−39	−7			+15	+30
	Kenya	−34	−19			+28	+20		
	Madagascar	−13	−15	−16	−26	+21	+29	+15	+8
	Rwanda	−54	−38			+32	+18		
	Tanzania	−37	−17	−23	−29	+31	+31	−2	−13
	Uganda			−43	−44			+2	+4
	Zambia			−55	−51			+14	−2
EAST	Total	−21	−17	−27	−31	+23	+28	+10	+6
Africa	Total	−20	−31	−23	−28	+14	−2	+10	+6

Empty spaces mean no simulations were conducted, which in most cases means the combination is absent. For example in Egypt almost all rice is irrigated rice in the main season, so no values for rainfed rice or for irrigated rice in the “off” season are shown. Similar tables for the all four scenarios (RCP2.6, 4.5, 6.0 and 8.5) are presented in the Tables S3–S6.

## 4 DISCUSSION

### 4.1 Main outcomes

This study is the first comprehensive study that addresses the impact of climate change on rice productivity in Africa. Overall, yield decline is found in all scenarios if farmers continue using the current varieties. Small yield increases are predicted if farmers adopt varieties that have a higher temperature sum, thus adapting to shortening of the growing duration induced by temperature increases. Two main causes of yield decline were identified:

1. Shortening growing period in all growing environments across Africa, and
2. Decrease in assimilation, but only in the hottest environment, i.e. irrigated rice cultivation in inland West‐Africa during the hot dry season.

Previous studies by Matthews et al. (1995, 1997) identified shortening growing periods (same as this study) and increasing spikelet sterility (contrary to this study) as the two main causes of future yield decline in Asia. This has spurred much research on heat induced sterility and so called “critical temperatures”. However, some recent studies still look at maximum air temperatures (Gourdji, Sibley, & Lobell, 2013), not accounting for transpirational cooling and early flowering. And no study so far has identified reduced assimilation at extreme temperatures as a possible cause of future rice yield reductions. Identifying these underlying mechanisms is relevant for breeders when screening for heat tolerant breeding material and can help formulate more specific follow‐up research questions.

### 4.2 West Africa: research needs

The way in which effects of CO_2_ and temperature are modelled can have large effects on model outcomes in climate change impact assessments (Li et al., 2015). (Adam, Van Bussel, Leffelaar, Van Keulen, & Ewert, 2011) and (Li et al., 2015) showed that the assumption of a constant radiation use efficiency is not realistic and that temperature effects need to be included. Two model approaches include such temperature effects (i) the simpler Light Response Curve photosynthesis model multiplied with a temperature function (LRC approach) or (ii) the Farquhar‐von Caemmerer‐Berry (FvCB approach) biochemical model of leaf photosynthesis (Farquhar, Von Caemmerer, & Berry, 1980). Li et al. (2015) found large variation in simulated biomass as a function of different crop growth models with different leaf photosynthesis submodels, but did not find systematic differences between LRC and FvCB based crop growth models. ORYZA2000 uses the LRC approach, in which the light response curve is multiplied with a trapezoid temperature response function (Figure 3). The origins of this function are unclear and the function has not been changed or tested since its’ implementation in the 1990s. Notably, this trapezoid temperature response function uses daily air temperature as the explanatory (*x*) variable. For the hot semiarid environment of irrigated dry season rice the possibility cannot be ruled out that, in analogy with the heat sterility case, increased transpirational cooling at higher temperatures and high vapour pressure deficit (Julia & Dingkuhn, 2013; Van Oort et al., 2014) acts as a natural adaptive mechanism allowing the plant to keep up photosynthesis in extremely hot climates with ample irrigation to sustain the transpiration flow. However, such strong transpirational cooling could also lead to lower intercellular leaf CO_2_ concentrations which could in theory lead to a decreasing photosynthesis rate (Leuning, 1995; Ogee, Brunet, Loustau, Berbigier, & Delzon, 2003; Wang & Leuning, 1998; Yin & Van Laar, 2005). Thus much uncertainty still remains. Albeit the existence of different leaf photosynthesis models, very limited experimental research has been conducted on rice photosynthesis in the extremely high (>40°C) temperature range. The single exception we are aware of is the study by (Stuerz, Sow, Muller, Manneh, & Asch, 2014) who measured in the hot (semi) arid delta of the Senegal river and who did not find any photosynthesis reductions even in the hottest part of the year. Closer analysis of temperatures in the study site of Stuerz et al. shows that there, temperatures were still just below the range where according to the ORYZA2000 model photosynthesis would be severely reduced (Figure 3). No rice leaf photosynthesis measurements have ever been reported from hotter environments, such as the inland dry season irrigated rice in countries like Mali, Niger, northern Benin and northern Nigeria. For these countries we are extrapolating with our model into a temperature range where models have hitherto not been tested, which makes our projections more uncertain. The uncertainties reported here probably also apply to other rice crop growth models (Li et al., 2015), as the root of the problem is the same: a lack of testing and lack of experimental data from extremely hot environments. Rice models would benefit from testing, comparison and improvement in such environments and testing across multiple environments. Our paper shows the relevance of such research and identifies target areas for such research.

Interestingly and in contradiction with previous studies (Matthews et al., 1995, 1997), the use of a new heat sterility model suggests that heat sterility in the future will hardly increase and not really become a problem. We note this is a preliminary conclusion. Heat sterility validations have been presented for the cooler delta and middle valley region of the Senegal river (Van Oort, De Vries et al., 2015), which is hot and dry but still cooler than in the inland dry season irrigated rice systems of West Africa, along the Niger river basin and the Benue river basin, where heat sterility models have to date not yet been tested. Parts of the new heat sterility model are still quite uncertain, especially the part predicting peak flowering time (Julia & Dingkuhn, 2012). This suggests also a need for more empirical and modelling research on heat sterility models.

### 4.3 East Africa: opportunities

According to von Liebig's law of the minimum (De Wit, 1992), crop production is constrained by the most limiting resource. Resources include light, atmospheric CO_2_ concentration, soil nutrient supply and water supply. Our simulations suggest that yield increases are possible in most of East Africa, caused by more favourable temperatures and increasing CO_2_ concentrations. According to von Liebig's law, these yield increases will only materialize if CO_2_ is more limiting than water and nutrient supply. In line with this, our simulations showed larger yield increases in the East African irrigated systems than in the rainfed systems, because there during parts of the growing season water is more limiting than CO_2_. This suggests that to benefit from climate change, East African countries will need to improve water management. The same rationale applies to nutrient supply: to sustain projected yield increases, more nutrient uptake will be needed, while soil fertility is low in much of Africa (Haefele, Nelson, & Hijmans, 2014) and fertilizer application levels are also often low. If water and nutrient management are not improved, then yields will increase only little, or not increase.

Lobell (2014) and Guan, Sultan, Biasutti, Baron, and Lobell (2017) argued that many technological interventions are as useful now as in the future and that they should therefore not be mistaken for adaptation options to climate change. They proposed a method to compute how much a technology contributes as a climate change adaptation option, by accounting for how much it would already contribute in the current climate. We applied the framework developed by (Guan et al., 2017) in Madagascar, because Madagascar is by far the largest rice producer in East Africa and because also for Madagascar our analysis suggested large yield increases (Table 5). We applied Guan's framework to two “technologies”: (i) conversion of rainfed land to irrigated land and (ii) choice of varieties with a higher temperature sum (“unchanged duration”), thus off‐setting natural shortening of the growing period due to temperature rise. Guan's framework defines four production *A*−*D* situations:

*A* = Yield with a technology that gives higher yields in the current climate; *B* = Yield without this technology, in the current climate; *C* = Yield with a technology that gives higher yields, future climate; *D* = Yield without this technology, in the future climate.

From these two adaptation impacts can be calculated (Guan et al., 2017):

(2) (A−B)/B=Impact in current climate

(3) [(C−D)−(A−B)]/B=Impact as a climate change adaptation option

Thus, for example if a technology would increase yields by 1 t/ha in the current climate (*A*−*B*) and also 1 t/ha in the future climate (*C*−*D*), then from Equation (3) it follows that the net contribution as an adaptation option to climate change is zero. Results (Table 6) showed that for the current climate hardly any yield gains from converting rainfed lowland into irrigated lowland (+3%). Rainfed uplands would benefit strongly from more irrigation (+71%). These results suggest that with a high groundwater table (40 cm) as we assumed for rainfed lowland soils, rainfall in Madagascar is sufficient to obtain potential production levels. If farmers stay with current varieties, irrigation does not help as an adaptation option to climate change: irrigation alone has the same positive effect now and in the future. The combination of irrigation and adapted varieties (i.e. with unchanged duration) does act as a promising climate change adaptation option with projected yield gains of 6% (rainfed lowland) to 28% (rainfed upland).

**Table 6:** Rice yields in Madagascar in current and future climate, with and without adaptations

Climate	Variety	Rice growing environment^a^		Potential or water limited yield (t/ha) b	(1) impact in current climate, % (Equation (2))^c^	(2) impact as a climate change adaptation, % (Equation (3))^c^
Current (2000)	Current	Irrigated	A	8.6		
		Rainfed lowland	B	8.3	+3	
		Rainfed upland	B	5.0	+71	
RCP8.5 2070	Shorter duration (=current)	Irrigated	C	7.4		
		Rainfed lowland	D	7.3		−1
		Rainfed upland	D	3.9		−1
	Unchanged duration	Irrigated	C	10.5		
		Rainfed lowland	D	9.8		+6
		Rainfed upland	D	5.5		+28

a The management option considered is introduction of irrigation in rainfed lowland or rainfed upland.

b Potential or water limited yield means simulated yields unrestricted by nutrient deficiencies and unrestricted by biotic stresses (weeds, pests and diseases).

c Impacts are calculated from *A*−*D*. For example for the upland systems, introducing irrigation in the current climate could increase yields by (*A*−*B*)/*B* = (8.6*−*5.0)/5.0 = +71%. As a climate change adaptation option irrigation contributes [(*C*−*D*) − (*A*−*B*)]/*B* = [(7.4−3.9)−(8.6−5.0)]/5.0 = −1% and the −1% is probably just a rounding error, the likely impact is zero. In rainfed upland systems the combination of irrigation and adapted varieties (unchanged duration) contributes as a climate change adaptation option [(10.5−5.5)−(8.6−5.0)]/5.0 = +28%.

The results raise the question whether in the current climate longer duration varieties might also be a good idea (Lobell, 2014). There are three arguments against doing so in this particular case. Firstly, a longer growing period would probably just lengthen the vegetative phase of rice growth, which is often already quite long in the cooler environment of Madagascar. In such situations, longer duration for a sink limited crop would give little yield gain. Secondly, there may be economic objections against longer duration varieties. If for example a variety with 180 vs. 150 days has a potential yield of 9 vs. 8 t/ha, then the marginal yield gain is 12.5% (=(9−8)/8) and the marginal increase in duration is 20% (=(180−150)/150). Since production costs (irrigation, weeding) are often strongly related to crop duration, a longer duration may not be profitable (Senegalese farmers for this reason reported preferring short duration varieties over available higher yielding medium duration varieties, see Van Oort et al., 2016). Thirdly, there is the issue of farm planning. Farmers in Madagascar often grow two crops per year, with a nonrice crop in the winter period (Harvey et al., 2014; Naudin et al., 2015). Labour availability and length of the growing period of the other crop may be a reason for not wanting to grow varieties with a longer duration, because this might create logistic problems. For these reasons, we opted not to explore the scenario of adaption of longer duration varieties in the current climate.

### 4.4 Uncertainties

A number of uncertainties were identified in this study. Firstly the leaf photosynthesis temperature response which we discussed above. Secondly the uncertainty about cold sterility (see our discussion in 2.2.2). A physiologically sufficiently sophisticated model would not require applying different parameters for the same variety in different regions such as we did in this study. Further empirical and modelling work is needed in this regard. Thirdly, the uncertainty caused by not accounting for soil fertility in our modelling work. We assumed the response to climate change would be similar under low and high soil fertility conditions. Recent research by Guan et al. (2017) showed in another crop that this may indeed be a plausible assumption. If projected climate change impact is negative then there would be no interaction with soil fertility, because less nutrients are demanded from the soil, so soil nutrient content does not matter. On the other hand if climate change impact shows increasing potential yields then more nutrients will be demanded from the soil. This extra nutrient uptake will be more easily met on the more fertile soils or if additional fertilizer is applied. In this scenario climate change impact would be positive only on fertile soils and be neutral on infertile soils, a three way positive interaction between soil fertility, CO_2_ fertilization and crop yield. Investigation of soil fertility interactions was impossible because it would require high resolution soil fertility mapping and validation of a rice model for nutrient balances, something which was beyond the scope of this paper. Clearly studying such interactions would be most relevant for East Africa, where our simulations show increasing potential yields. The fourth uncertainty is that of possible change in rainfall patterns. Large uncertainty exists about future changes in precipitation (Giannini et al., 2008; Lobell & Burke, 2008; Lobell et al., 2008). No scenario data are available with projections of changes in within‐season daily rainfall patterns. This comes on top of our assumptions on groundwater depth, for which also no high (spatial and temporal) resolution African datasets are available. Altogether we deem these uncertainties too large to allow for meaningful analyses of their effects on future rainfed rice yields. Future increased rainfall intensity might also cause increased flood risk (Aich et al., 2016), which we could not model because ORYZA2000 has no component simulating damage due to flooding and because of lack of high resolution rice maps along river systems in Africa.

Relatively much awareness exist of the above four uncertainties in the scientific community. There are two uncertainties of which we are aware but for which we are even more uncertain than those listed above. These are future effects of climate change on salinity and on cyclone frequency. It is known that climate change will lead to sea level rise which will affect mangrove rice systems in coastal zones, which according to Balasubramanian et al. (2007) represent 9% of Africa's rice area. The number of unknowns is too large to allow for quantitative estimation of how far sea water intrusion will increase and how this will affect mangrove rice areas. Rice in Africa is only affected by cyclones in Madagascar, one of the largest rice producers of Africa. According to (Harvey et al., 2014) cyclones have in the last 5 years affected 51% of all farmers in Madagascar, causing severe yield losses. Cyclone frequency worldwide is in general expected to increase with climate change, but how much frequency will increase in Madagascar is still very uncertain (Mendelsohn, Emanuel, Chonabayashi, & Bakkensen, 2012). Thus there are a number of additional negative climate change effects that can be anticipated but for which the magnitude of changes as well as the possible impacts are still very uncertain.

### 4.5 Synthesis

Overall, negative impacts of climate change on rice yields in Africa are shown in all scenarios if farmers stay with current varieties. Predominantly positive effects are observed if farmers adopt varieties with a higher temperature sum, keeping pace with shortening of the growing duration due to temperature. With this adaptation option, rice yields in irrigated environments in East Africa could increase (around +25% in the most extreme scenario RCP8.5, from 2000 to 2070) and they will increase less in rainfed rice environments. Irrigated rice yields in the hot dry season of West Africa will reduce significantly due to reduced photosynthesis. For East Africa to benefit from climate change, improved water management and possibly also soil fertility management will be needed in combination with gradually replacing current varieties with varieties with a higher temperature sum. In West Africa, more research is required to improve our knowledge on photosynthesis processes during extreme temperatures and research is needed on adaptation options for rice farmers such as shifting sowing dates more into the cold dry season.
