# Supplementary material for: Impacts of climate change on rice production in Africa and causes of simulated yield changes
Source: Glob Chang Biol. 2017 Dec 12;24(3):1029–45. doi: 10.1111/gcb.13967 (PMC5836867; doi:10.1111/gcb.13967)
Supplement: Supplementary file 1 [file GCB-24-1029-s001.docx]

# Supporting information

The Supporting Information contains five subsections. Sub-section 1.1 provides background on the climate change data. Sub-section 1.2 describes in detail how leaf level assimilation is simulated in ORYZA2000 and how it is driven by climatic factors. Section 1.3 lists the assumed soil parameters. Section 1.4 shows yield changes (2000-2070) per country, environment and scenario, with and without adaptation, with one table per scenario. Section 1.5 lists references cited in this Supporting Information.

## Climate change scenarios

The full set of maps of changes per season and scenario for precipitation, T_max_ and T_min_ is available at <https://www.researchgate.net/project/Rice-and-Cimate-Change> (Zwart, 2016). Table S1 lists the General Circulation Models (GCMs) used in this study for each of the Representative Concentration Pathways (RCPs).

**Table S1: General Circulation Models (GCMs) utilized in this study for each of the Representative Concentration Pathways (RCPs) as defined by the IPCC.**

| GCM | RCP2.6 | RCP4.5 | RCP6.0 | RCP8.5 |
| --- | --- | --- | --- | --- |
| bcc_csm1_1 | - | x | x | x |
| bcc_csm1_1_m | - | x | x | x |
| bnu_esm | x | x | - | x |
| cccma_canesm2 | x | x | - | x |
| cesm1_bgc | - | x | - | x |
| cesm1_cam5 | x | x | x | x |
| csiro_access1_0 | - | x | - | x |
| csiro_access1_3 | - | x | - | x |
| csiro_mk3_6_0 | x | x | x | x |
| fio_esm | x | x | x | x |
| ec_earth | - | - | - | x |
| gfdl_cm3 | x | x | x | x |
| gfdl_esm2g | x | x | x | x |
| gfdl_esm2m | x | x | x | x |
| giss_e2_h | x | - | x | x |
| giss_e2_h_cc | - | x | - | - |
| giss_e2_r | x | x | x | x |
| giss_e2_r_cc | - | x | - | - |
| inm_cm4 | - | x | - | x |
| ipsl_cm5a_lr | x | x | x | x |
| ipsl_cm5a_mr | x | x | - | x |
| lasg_fgoals_g2 | x | x | - | x |
| miroc_esm | x | x | x | x |
| miroc_esm_chem | x | x | x | x |
| miroc_miroc5 | x | x | x | x |
| mohc_hadgem2_cc | - | x | - | x |
| ipsl_cm5b_lr | - | - | - | x |
| mohc_hadgem2_es | x | x | x | x |
| mpi_esm_lr | x | x | - | x |
| mpi_esm_mr | x | - | - | x |
| mri_cgcm3 | x | x | x | x |
| ncar_ccsm4 | x | x | x | x |
| ncc_noresm1_m | x | x | x | x |
| nimr_hadgem2_ao | x | x | x | x |
| TOTAL | 25 | 30 | 19 | 32 |

## Gross leaf assimilation in ORYZA2000

ORYZA2000 simulates leaf photosynthesis at 3 times per day (at different temperatures and radiation levels early morning, midday and late afternoon) and at three canopy depths, for sunlit and shaded leaves, and then integrates over the canopy and the day to obtain the daily gross assimilation rate (Bouman *et al.*, 2001). This integration is of lesser interest here. Here we describe how leaf level assimilation is simulated. The following description of assimilation is entirely based on equations and parameters in the ORYZA2000 book (Bouman *et al.*, 2001). Assimilation in ORYZA2000 increases with intercepted radiation following the well-known asymptotic light response curve (de Wit, 1978) . Figure S1a shows the light response curve at 30oC daytime air temperature with leaves having a 1.5 g /m2 leaf N content. The light response curve (Fig. S1a) has an initial light use efficiency (slope at x = 0 in Fig. S1a) which is temperature dependent (Fig. S1b) and a maximum daily assimilation rate (asymptote in in Fig. S1a) which depends on leaf nitrogen content (Fig. S1c) and is multiplied with a unitless CO_2_ effect (Fig. S1d) and temperature effect (Fig. S1e). If the assumption is made that nitrogen is non-limiting, as we did in this study, leaf nitrogen content is assumed to be dependent on development stage only (Fig. S1f) and assumed to have a nitrogen extinction within the canopy with extinction coefficient k = 0.4. The two temperature effects in the model are both with daytime average temperature which is calculated as 0.75*T_max_ +0.25*T_min_ (daily maximum and minimum air temperature). Thus when simulating for a specific time early in the morning the gross assimilation will be calculated with intercepted radiation levels at that that specific time of the day, using the using a light response curve typical for that day (which depends on daytime average temperature, through Fig. S1b and S1e). The combined effect of light, nitrogen, CO_2_ and temperature is shown in Figures S1g and S1h. Comparing figures S1g and S1h we can see:

1. CO_2_ fertilisation increases assimilation
2. According to this model, assimilation rate drops rapidly as a function of air temperature when daytime temperature is above 37^o^C

The temperature response model therefore does not account for possible effects of atmospheric CO_2_ on optimum temperature and it does not account for transpirational cooling of the leaves as driven by vapour pressure deficit and effect of vapour pressure deficit on the intercellular CO_2_ concentration (Leuning, 1995, Ogee *et al.*, 2003, Wang & Leuning, 1998, Yin & Laar, 2005)

| 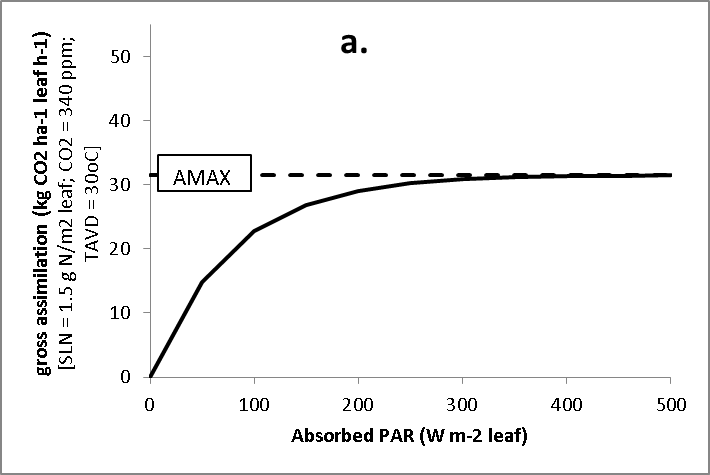 | 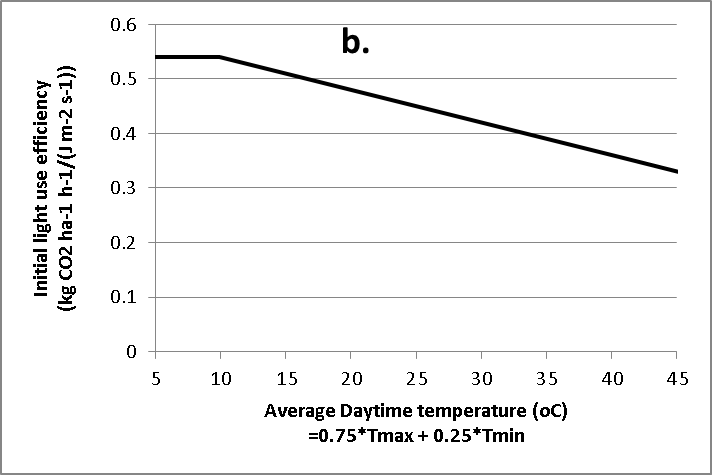 |
| --- | --- |
| 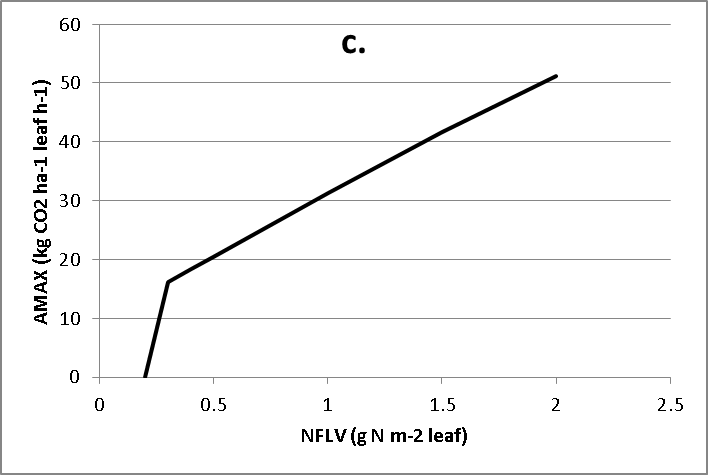 | 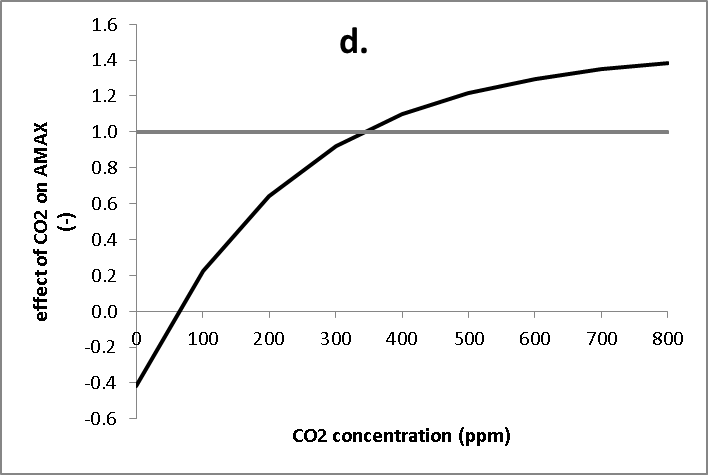 |
| 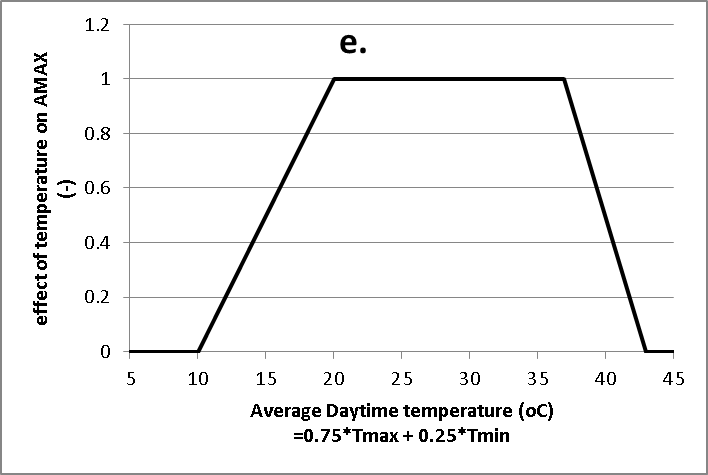 | 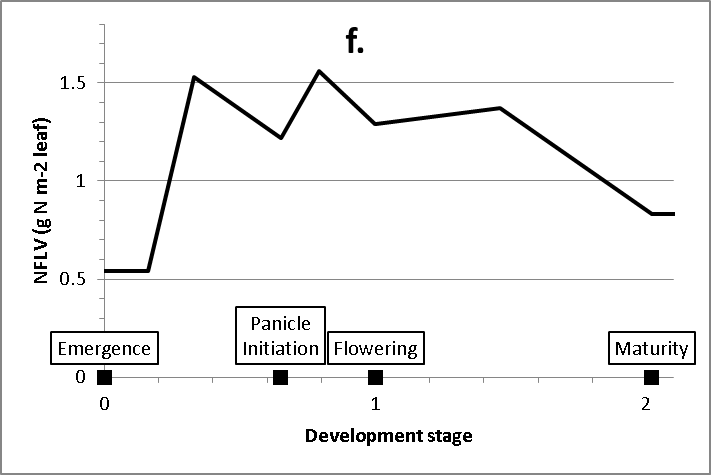 |
| 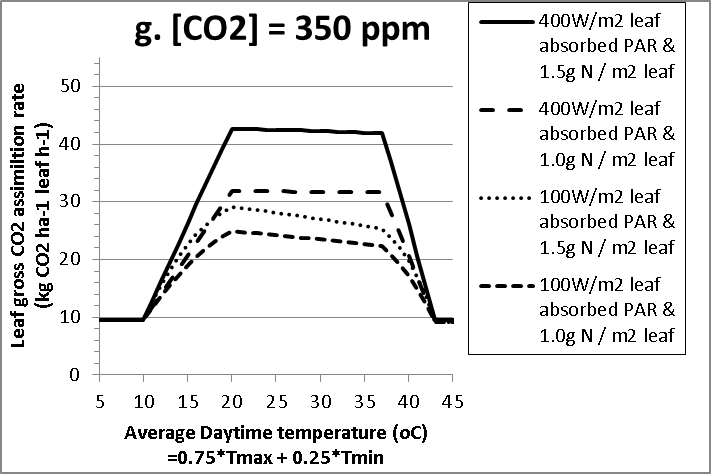 | 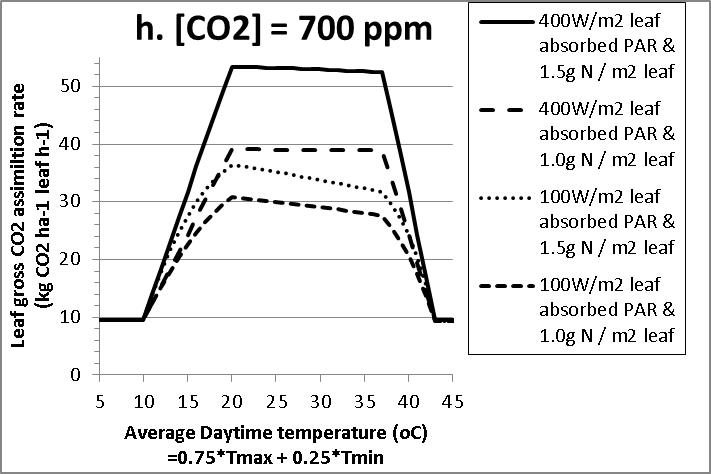 |

**Figure S1. Leaf gross assimilation as affected by (a) light interception, (b) temperature at low light conditions (c), leaf nitrogen (d) CO2, (e) Temperature. (f) Leaf nitrogen content at different development stages. (g) and (h) show combined effects of light, N, CO2 and temperature.**

## Soil parameters

Rainfed rice yields depend strongly on groundwater depth and percolation losses (Boling *et al.*, 2007, Bouman *et al.*, 2007, Bouman *et al.*, 1994, Wopereis *et al.*, 1994). Rainfed lowland rice generally has high groundwater levels and low percolation rates, rainfed upland generally has freely draining soils, i.e. with deep groundwater levels and high percolation rates. Note that this is a continuum: as one moves from the highest landscape positions (upland rice) to the lowest (lowland rice) one moves through the hydromorphic slopes with increasingly shallow groundwater depth. No large scale high resolution (spatial and temporal) groundwater datasets are available for Africa. We therefore made two assumptions with regards to a typical lowland soil and a typical upland soil. Table S2 lists the assumed parameters. Note that in our simulations, actual water levels can be higher than the superimposed minimum depth of 40cm in lowlands: if daily rainfall exceeds daily percolation plus evapotranspiration and if the soil is saturated, and if there are bunds, then a ponded layer will develop (flooded field), something which the ORYZA2000 model can also simulate. Note also that percolation rates shown apply only in case of standing water. If water supply is less than water demand (ET) then percolation will be simulated based on soil hydraulic conductivity.

**Table S2. Assumed soil parameters for a lowland and upland soil**

| Soil parameters^1^ | Lowland | Upland |
| --- | --- | --- |
| Soil texture | Clayey | Sandy |
| Saturated moisture content *θ_s_* (m3 m-3)^2^ | 0.57 | 0.39 |
| Van Genuchten parameter^2^ *α* (cm-1) | 0.0205 | 0.0321 |
| Van Genuchten parameter *l* (-) | 40.7 | 60.9 |
| Van Genuchten parameter *n* (-) | 1.2848 | 2.163 |
| Van Genuchten parameter *θ_r_* (-) | 0.27 | 0.04 |
| Van Genuchten parameter k_s_ ^2^ (cm/day) | 10.789 | 99.77 |
| Bund height (mm)^3^ | 250 | 0 |
| Groundwater depth (cm) | 40 | 1000 |
| Percolation rate (mm/day)^4^ | 3.7 | 240.3 |
| Puddling^5^ | No | No |
| Initial moisture content: Field Capacity | 0.562 | 0.375 |

^1^ units are consistent with those used in (Bouman *et al.*, 2001). We are aware it looks a bit awkward that some are in meters, some in centimetres and some in millimetres

^2^ (van Genuchten, 1980) equations:

$S=\frac{\theta-\theta_{r}}{\left( \theta_{s}-\theta_{r} \right)}=\left[ 1+{|\alpha|}^{n} \right]^{-m}$ with *m* = 1-1/*n.*

$k(S)=k_{s}S^{l}\left[ 1-{(1-S^{l/m})}^{m} \right]^{2}$

Where S(-) is the degree of saturation, θ (-) is soil volumetric water content and k(S) is the hydraulic conductivity (cm/day) at S and parameters are de

^3^ Bund height 0mm for upland soils means there are no bunds, which is the common situation for most upland rice in Africa

^4^ We chose the option in ORYZA2000 to simulate with a fixed percolation in cases of standing (above ground) water. The high number for the upland soil percolation rate (99.77cm/day) implies that any standing (pond) water would immediately drain. Without standing water downward flow is calculated based on hydraulic conductivity (Bouman *et al.*, 2001) which is less than the percolation rate and which is calculated with the van Genuchten equation for hydraulic conductivity

^5^ Few farmers in Africa practice puddling, even in irrigated systems

## RCP scenarios 2000-2070 by country and environment

Tables S3-S6 in the following pages show the simulated yield changes in the four RCP scenarios 2.6, 4.5, 6.0 and 8.5, split out by country and environment, without adaptation (shorter cycle) and with adaptation (same cycle). These tables show the same basic pattern (yield decline in off-season irrigated rice in West –Africa landlocked countries and yield increases in East African highlands). The magnitude of these yield changes is larger as me move from the most moderate scenario 2.6 to the most extreme 8.5. The coloured cells (heat map) help visualise the magnitude of changes.

**Table S3. Simulated rice yield changes between 2000 to 2070 for RCP 2.6**

|  |  | Rice yield changes 2000 to 2070. RCP 2.6 | | | | | | | |
| --- | --- | --- | --- | --- | --- | --- | --- | --- | --- |
|  |  | Shorter duration | | | | Unchanged duration | | | |
|  |  | Irrigated | | Rainfed | | Irrigated | | Rainfed | |
| Region | Country | Main season (wet) | Off season (dry) | Low-land | Upland | Main season (wet) | Off season (dry) | Low-land | Upland |
| WEST | Benin | -0.03 | -0.18 | -0.01 | -0.02 | 0.06 | -0.08 | 0.07 | 0.08 |
|  | Burkina Faso | 0.01 | -0.07 | -0.01 | 0.03 | 0.08 | 0.00 | 0.07 | 0.14 |
|  | Cote D'Ivoire | -0.02 |  | -0.04 | -0.08 | 0.06 |  | 0.04 | 0.03 |
|  | Cameroon | 0.01 | -0.17 |  |  | 0.07 | -0.04 |  |  |
|  | Ghana | -0.06 | -0.06 | -0.05 | -0.09 | 0.05 | 0.00 | 0.05 | 0.07 |
|  | Gambia | -0.08 | -0.08 | 0.12 | 0.25 | 0.04 | 0.02 | 0.04 | 0.08 |
|  | Mali | -0.09 | -0.27 | 0.03 | 0.02 | 0.01 | -0.18 | 0.03 | 0.02 |
|  | Mauritania | -0.03 | -0.08 |  |  | 0.06 | 0.00 |  |  |
|  | Niger | -0.07 | -0.13 |  |  | 0.02 | -0.14 |  |  |
|  | Nigeria | -0.06 | -0.10 | -0.09 | -0.09 | 0.07 | 0.00 | 0.05 | 0.02 |
|  | Senegal | -0.04 | -0.04 |  |  | 0.04 | 0.02 |  |  |
| WEST | Total | -0.05 | -0.11 | -0.04 | -0.06 | 0.05 | -0.04 | 0.05 | 0.04 |
| NORTH | Egypt | -0.10 |  |  |  | 0.01 |  |  |  |
| EAST | Ethiopia |  |  | 0.59 | 0.76 |  |  | 0.99 | 0.69 |
|  | Kenya | -0.13 | -0.08 |  |  | 0.06 | 0.06 |  |  |
|  | Madagascar | -0.07 | -0.06 | -0.10 | -0.14 | 0.06 | 0.08 | 0.04 | -0.02 |
|  | Rwanda | -0.17 | -0.16 |  |  | 0.09 | 0.06 |  |  |
|  | Tanzania | -0.13 | -0.06 | -0.03 | -0.01 | 0.13 | 0.09 | 0.02 | -0.03 |
|  | Uganda |  |  | -0.20 | -0.28 |  |  | 0.03 | -0.03 |
|  | Zambia |  |  | -0.24 | -0.20 |  |  | 0.05 | 0.03 |
| EAST & NORTH | Total | -0.09 | -0.07 | -0.11 | -0.15 | 0.07 | 0.08 | 0.06 | 0.01 |
| Total | Countries simulated | -0.07 | -0.09 | -0.07 | -0.10 | 0.06 | 0.01 | 0.05 | 0.03 |

**Table S4. Simulated rice yield changes between 2000 to 2070 for RCP 4.5**

|  | | | |  | | |  |  | |  | |  | |  | |  |
| --- | --- | --- | --- | --- | --- | --- | --- | --- | --- | --- | --- | --- | --- | --- | --- | --- |
| Scenario | 4_5 | Rice yield changes 2000 to 2070. RCP 4.5 | | | | | | | | | | | | | | |
| Year | 2070 | Shorter duration | | | | | | | Unchanged duration | | | | | | | |
|  |  | Irrigated | | | Rainfed | | | | Irrigated | | | | Rainfed | | | |
| Region | Country | Main season (wet) | Off season (dry) | | Low-land | Upland | | | Main season (wet) | | Off season (dry) | | Low-land | | Upland | |
| WEST | Benin | -0.12 | -0.44 | | -0.15 | -0.20 | | | 0.08 | | -0.26 | | 0.08 | | 0.00 | |
|  | Burkina Faso | -0.15 | -0.35 | | -0.26 | -0.23 | | | 0.08 | | -0.19 | | 0.02 | | 0.07 | |
|  | Cote D'Ivoire | -0.12 |  | | -0.13 | -0.25 | | | 0.10 | |  | | 0.06 | | 0.03 | |
|  | Cameroon | -0.02 | -0.35 | |  |  | | | 0.11 | | -0.13 | |  | |  | |
|  | Ghana | -0.18 | -0.27 | | -0.17 | -0.34 | | | 0.07 | | -0.10 | | 0.06 | | 0.00 | |
|  | Gambia | -0.24 | -0.23 | | 0.19 | 0.50 | | | 0.03 | | -0.05 | | 0.10 | | 0.15 | |
|  | Mali | -0.24 | -0.65 | | -0.06 | -0.12 | | | -0.03 | | -0.52 | | 0.03 | | -0.06 | |
|  | Mauritania | 0.01 | -0.15 | |  |  | | | 0.13 | | 0.00 | |  | |  | |
|  | Niger | -0.20 | -0.32 | |  |  | | | -0.04 | | -0.35 | |  | |  | |
|  | Nigeria | -0.23 | -0.28 | | -0.22 | -0.24 | | | 0.05 | | -0.09 | | 0.06 | | -0.01 | |
|  | Senegal | -0.01 | -0.11 | |  |  | | | 0.10 | | 0.02 | |  | |  | |
| WEST | Total | -0.16 | -0.31 | | -0.16 | -0.22 | | | 0.05 | | -0.17 | | 0.05 | | 0.01 | |
| NORTH | Egypt | -0.13 |  | |  |  | | | 0.05 | |  | |  | |  | |
| EAST | Ethiopia |  |  | | 0.08 | 0.32 | | |  | |  | | 0.98 | | 0.79 | |
|  | Kenya | -0.29 | -0.15 | |  |  | | | 0.12 | | 0.12 | |  | |  | |
|  | Madagascar | -0.11 | -0.13 | | -0.15 | -0.22 | | | 0.13 | | 0.18 | | 0.09 | | 0.02 | |
|  | Rwanda | -0.43 | -0.36 | |  |  | | | 0.15 | | 0.11 | |  | |  | |
|  | Tanzania | -0.29 | -0.15 | | -0.13 | -0.12 | | | 0.19 | | 0.17 | | 0.00 | | -0.11 | |
|  | Uganda |  |  | | -0.36 | -0.42 | | |  | |  | | -0.01 | | -0.06 | |
|  | Zambia |  |  | | -0.39 | -0.40 | | |  | |  | | 0.10 | | -0.01 | |
| EAST & NORTH | Total | -0.17 | -0.16 | | -0.21 | -0.26 | | | 0.14 | | 0.16 | | 0.08 | | 0.02 | |
| Total | Countries simulated | -0.16 | -0.24 | | -0.18 | -0.24 | | | 0.09 | | -0.02 | | 0.07 | | 0.02 | |

**Table S5. Simulated rice yield changes between 2000 to 2070 for RCP 6.0**

|  |  | Rice yield changes 2000 to 2070. RCP 6.0 | | | | | | | |
| --- | --- | --- | --- | --- | --- | --- | --- | --- | --- |
|  |  | Shorter duration | | | | Unchanged duration | | | |
|  |  | Irrigated | | Rainfed | | Irrigated | | Rainfed | |
| Region | Country | Main season (wet) | Off season (dry) | Low-land | Upland | Main season (wet) | Off season (dry) | Low-land | Upland |
| WEST | Benin | -0.10 | -0.39 | -0.14 | -0.18 | 0.10 | -0.23 | 0.10 | 0.01 |
|  | Burkina Faso | -0.13 | -0.26 | -0.23 | -0.19 | 0.10 | -0.11 | 0.05 | 0.11 |
|  | Cote D'Ivoire | -0.09 |  | -0.10 | -0.20 | 0.13 |  | 0.09 | 0.06 |
|  | Cameroon | -0.03 | -0.30 |  |  | 0.12 | -0.09 |  |  |
|  | Ghana | -0.15 | -0.23 | -0.14 | -0.32 | 0.10 | -0.06 | 0.09 | 0.03 |
|  | Gambia | -0.19 | -0.13 | 0.23 | 0.50 | 0.07 | 0.02 | 0.14 | 0.20 |
|  | Mali | -0.19 | -0.55 | -0.02 | -0.07 | 0.01 | -0.40 | 0.06 | 0.02 |
|  | Mauritania | 0.06 | -0.08 |  |  | 0.17 | 0.06 |  |  |
|  | Niger | -0.17 | -0.29 |  |  | -0.02 | -0.32 |  |  |
|  | Nigeria | -0.22 | -0.24 | -0.20 | -0.22 | 0.06 | -0.06 | 0.08 | 0.01 |
|  | Senegal | 0.03 | -0.04 |  |  | 0.14 | 0.08 |  |  |
| WEST | Total | -0.13 | -0.26 | -0.13 | -0.19 | 0.08 | -0.12 | 0.08 | 0.04 |
| NORTH | Egypt | -0.10 |  |  |  | 0.08 |  |  |  |
| EAST | Ethiopia |  |  | 0.22 | 0.39 |  |  | 1.07 | 0.68 |
|  | Kenya | -0.23 | -0.12 |  |  | 0.15 | 0.15 |  |  |
|  | Madagascar | -0.08 | -0.10 | -0.11 | -0.18 | 0.15 | 0.20 | 0.12 | 0.06 |
|  | Rwanda | -0.33 | -0.27 |  |  | 0.18 | 0.14 |  |  |
|  | Tanzania | -0.24 | -0.11 | -0.09 | -0.09 | 0.23 | 0.19 | 0.04 | -0.02 |
|  | Uganda |  |  | -0.33 | -0.38 |  |  | 0.02 | 0.01 |
|  | Zambia |  |  | -0.37 | -0.37 |  |  | 0.12 | 0.01 |
| EAST & NORTH | Total | -0.13 | -0.12 | -0.17 | -0.22 | 0.17 | 0.19 | 0.11 | 0.06 |
| Total | Countries simulated | -0.13 | -0.19 | -0.15 | -0.20 | 0.12 | 0.02 | 0.09 | 0.05 |

**Table S6. Simulated rice yield changes between 2000 to 2070 for RCP 8.5**

|  |  | Rice yield changes 2000 to 2070. RCP 8.5 | | | | | | | |
| --- | --- | --- | --- | --- | --- | --- | --- | --- | --- |
|  |  | Shorter duration | | | | Unchanged duration | | | |
|  |  | Irrigated | | Rainfed | | Irrigated | | Rainfed | |
| Region | Country | Main season (wet) | Off season (dry) | Low-land | Upland | Main season (wet) | Off season (dry) | Low-land | Upland |
| WEST | Benin | -0.13 | -0.59 | -0.21 | -0.25 | 0.13 | -0.41 | 0.13 | 0.11 |
|  | Burkina Faso | -0.23 | -0.49 | -0.31 | -0.28 | 0.07 | -0.33 | 0.07 | 0.12 |
|  | Cote D'Ivoire | -0.13 |  | -0.13 | -0.28 | 0.17 |  | 0.11 | 0.08 |
|  | Cameroon | -0.04 | -0.52 |  |  | 0.14 | -0.31 |  |  |
|  | Ghana | -0.20 | -0.36 | -0.18 | -0.37 | 0.13 | -0.16 | 0.11 | 0.07 |
|  | Gambia | -0.25 | -0.30 | 0.29 | 0.85 | 0.06 | -0.05 | 0.18 | 0.26 |
|  | Mali | -0.33 | -0.80 | -0.09 | -0.17 | -0.07 | -0.70 | 0.04 | 0.00 |
|  | Mauritania | 0.07 | -0.14 |  |  | 0.21 | 0.02 |  |  |
|  | Niger | -0.29 | -0.45 |  |  | -0.10 | -0.48 |  |  |
|  | Nigeria | -0.30 | -0.42 | -0.25 | -0.27 | 0.06 | -0.18 | 0.11 | 0.04 |
|  | Senegal | 0.04 | -0.10 |  |  | 0.18 | 0.06 |  |  |
| WEST | Total | -0.20 | -0.43 | -0.18 | -0.25 | 0.07 | -0.27 | 0.10 | 0.07 |
| NORTH | Egypt | -0.19 |  |  |  | 0.06 |  |  |  |
| EAST | Ethiopia |  |  | -0.04 | 0.29 |  |  | 1.08 | 0.88 |
|  | Kenya | -0.35 | -0.19 |  |  | 0.20 | 0.20 |  |  |
|  | Madagascar | -0.14 | -0.16 | -0.17 | -0.24 | 0.20 | 0.26 | 0.14 | 0.08 |
|  | Rwanda | -0.58 | -0.37 |  |  | 0.22 | 0.18 |  |  |
|  | Tanzania | -0.38 | -0.18 | -0.23 | -0.29 | 0.26 | 0.26 | 0.00 | -0.15 |
|  | Uganda |  |  | -0.44 | -0.47 |  |  | 0.01 | 0.02 |
|  | Zambia |  |  | -0.55 | -0.55 |  |  | 0.14 | 0.10 |
| EAST & NORTH | Total | -0.21 | -0.18 | -0.26 | -0.30 | 0.21 | 0.25 | 0.11 | 0.08 |
| Total | Countries simulated | -0.21 | -0.31 | -0.22 | -0.27 | 0.13 | -0.04 | 0.11 | 0.07 |

## References

Boling AA, Bouman BaM, Tuong TP, Murty MVR, Jatmiko SY (2007) Modelling the effect of groundwater depth on yield-increasing interventions in rainfed lowland rice in Central Java, Indonesia. *Agricultural Systems,* **92**, 115-139.

Bouman BAM, Humphreys E, Tuong TP, Barker R (2007) Rice and water. *Advances in Agronomy, Vol 92,* **92**, 187-237.

Bouman BAM, Kropff MJ, Tuong TP, Wopereis MCS, Ten Berge HFM, Van Laar HH (2001) *ORYZA2000: modeling lowland rice,* Los Baños, IRRI.

Bouman BAM, Wopereis MCS, Kropff MJ, Tenberge HFM, Tuong TP (1994) Water-Use Efficiency of Flooded Rice Fields .2. Percolation and Seepage Losses. *Agricultural Water Management,* **26**, 291-304.

De Wit CT (1978) *Simulation of assimilation, respiration and transpiration of crops,* New York, Wiley.

Leuning R (1995) A Critical-Appraisal of a Combined Stomatal-Photosynthesis Model for C-3 Plants. *Plant Cell and Environment,* **18**, 339-355.

Ogee J, Brunet Y, Loustau D, Berbigier P, Delzon S (2003) MuSICA, a CO2, water and energy multilayer, multileaf pine forest model: evaluation from hourly to yearly time scales and sensitivity analysis. *Global Change Biology,* **9**, 697-717.

Van Genuchten MT (1980) Closed-form equation for predicting the hydraulic conductivity of unsaturated soils. *Soil Science Society of America Journal,* **44**, 892-898.

Wang YP, Leuning R (1998) A two-leaf model for canopy conductance, photosynthesis and partitioning of available energy I: Model description and comparison with a multi-layered model. *Agricultural and Forest Meteorology,* **91**, 89-111.

Wopereis MCS, Bouman BAM, Kropff MJ, Tenberge HFM, Maligaya AR (1994) Water-Use Efficiency of Flooded Rice Fields .1. Validation of the Soil-Water Balance Model Sawah. *Agricultural Water Management,* **26**, 277-289.

Yin X, Laar HHV (2005) *Crop systems dynamics : an ecophysiological simulation model for genotype-by-environment interactions,* Wageningen, Wageningen Academic.

Zwart SJ, 2016. Projected climate conditions for rice production systems in Africa. AfricaRice GIS Report – 1. Africa Rice Center, Cotonou, Benin
